# Preferences for End-of-Life Care Among Patients With Terminal Cancer in China

**DOI:** 10.1001/jamanetworkopen.2022.8788

**Published:** 2022-04-25

**Authors:** Anli Leng, Elizabeth Maitland, Siyuan Wang, Stephen Nicholas, Kuixu Lan, Jian Wang

**Affiliations:** 1 School of Political Science and Public Administration, Shandong University, Qingdao, China; 2 Center for Health Preferences Research, Shandong University, Jinan, China; 3 School of Management, University of Liverpool, Liverpool, England; 4 Faculty of Business and Economics, University of Melbourne, North Melbourne, Victoria, Australia; 5 Australian National Institute of Management and Commerce, Australian Technology Park, Sydney, New South Wales, Australia; 6 Newcastle Business School, University of Newcastle, Newcastle, New South Wales, Australia; 7 Publicity Department, The Affiliated Hospital of Qingdao University, Shinan District, Qingdao, China; 8 Dong Fureng Institute of Economic and Social Development, Wuhan University, Beijing, China; 9 Center for Health Economics and Management at the School of Economics and Management, Wuhan University, Wuhan, China

## Abstract

**Importance:**

In China, little is known about end-of-life (EOL) care preferences of patients with terminal cancer. Understanding these patients’ treatment preferences is needed to improve patient-centered health care, better inform surrogates and medical staff about patient preferences, and enhance the quality of EOL care.

**Objective:**

To examine preferences for EOL care among patients with terminal cancer in China.

**Design, Setting, and Participants:**

In this survey study, patients older than 50 years who had terminal cancer were randomly selected from medical records at a single hospital in China. Data on patients’ EOL care preferences were collected by discrete choice experiment (DCE) from August to November 2018 and were analyzed from October 2020 to March 2021.

**Main Outcomes and Measures:**

The main outcome was patient preferences in EOL care, derived using a mixed logit model. Each DCE scenario described 6 attributes: hospitalization days, life extension, quality of life, adverse treatment events, place-of-death preference, and out-of-pocket costs. The marginal willingness to pay (WTP) in US dollars was estimated from regression coefficients.

**Results:**

Of 188 patients selected for the survey, 183 participated (97.3%). Among the respondents, the mean [SD] age was 61 [8.4] years, and 128 (69.8%) were male. Patients’ preferences for moderate increase in survival time, better quality of life, death at home, and lower out-of-pocket costs were significantly associated with their choices between treatment models. Extending life by 10 months (vs 4 months: β, 1.63; 95% CI, 0.81-2.44) and a better quality of life (very good vs poor: β, 1.79; 95% CI, 0.96-2.62) were the most important attributes to patients. The uptake rate for a treatment scenario increased by 61.6% when the quality of life improved from poor to very good, and when life extension increased from 4 months to 10, the uptake rate increased by 57.2%. The uptake increased by 12.5% when the place of death changed from hospital to home. However, it decreased by 31.4% when the costs increased to $21 174. The study found a WTP of $38 854 (95% CI, $19 468-$95 096) to improve quality of life from a poor to a very good level, substantially higher than the WTP for a life extension of 6 months ($35 308; 95% CI, $17 745-$80 279) or 1 year ($27 572; 95% CI, $16 389-$58 027) compared with the baseline scenario of a 4-month extension. Patients were willing to pay $8860 (95% CI, $621-$26 474) to die at home rather than in a hospital.

**Conclusions and Relevance:**

The findings suggest that in addition to extending life moderately for patients with terminal cancer, improving quality of life during EOL care and supporting home deaths may deserve greater attention. The findings also suggest that physicians and surrogates should ask about patients’ care preferences and better inform them of their choices to improve EOL care outcomes.

## Key Points

**Question:**

What are the preferences for end-of-life care among adult patients with terminal cancer in China?

**Findings:**

In this survey study using a discrete choice experiment among 183 Chinese patients older than 50 years with terminal cancer, patients preferences for a moderate increase in survival time, better quality of life, death at home, and lower out-of-pocket costs were significantly associated with choices between treatment models.

**Meaning:**

The findings suggest that to improve end-of-life outcomes, physicians and surrogates of patients with terminal cancer should ask about patients’ care preferences and better inform them of their choices.

## Teaser

This survey study investigates preferences for end-of-life care among adult patients with terminal cancer in China.

## Introduction

China’s National Cancer Center estimated that there were 4.3 million new cases of cancer in 2015, with the annual incidence increasing by approximately 3.9% during the past decade.^1^ On average, more than 10 000 people in China are diagnosed with cancer every day, or 7.5 people every minute.^1^ More than 2.8 million deaths from cancer occurred in China in 2015, accounting for almost one-third of all deaths from cancer worldwide.^2^ With China’s cancer mortality rate increasing by 2.5% a year,^3^ the number of people in need of end-of-life (EOL) care poses a substantial challenge to China’s public health system.

In China, the annual medical expenditures for malignant tumors exceed $36 billion.^4^ Accounting for approximately 40% of the total health care expenses for patients after they are diagnosed with cancer and mainly attributable to hospital costs, terminal cancer in China imposes a per capita expenditure of $13 572 for patients with cancer living in urban areas and $6510 for patients with cancer living in rural areas in the last 3 months of life.^5^ End-of-life health care costs impose a substantial economic burden on Chinese patients and their families, with more than 90% of families experiencing catastrophic EOL health expenditures^6^ and 32.8% of patients borrowing from family and friends to pay their medical expenses.^5^ Worries about money and physical pain, psychological pressure, and fear of death are associated with choice of treatment among patients with terminal cancer.^7^ In China, EOL quality is poor for most patients with terminal cancer partly owing to the overuse of hospital-based treatments and the low use of palliative care.^5^

An important prerequisite for improving the quality of patient-oriented EOL care is to understand patient preferences or patient care trade-offs including life extension, quality of life, place of care, and place of death. An Australian cross-sectional study found that most older and seriously ill inpatients preferred EOL care that maintained their quality of life compared with life extension.^8^ Patients with advanced prostate cancer in 2 London hospitals were willing to pay to reduce the burden of adverse events,^9^ and a Singapore study found that patients with advanced cancer were willing to pay to avoid severe pain, to die at home, and to not burden their families.^10^ Voogt et al^11^ found that Dutch patients who had cancer for less than 6 months were more inclined to prefer life extension treatments than were those with a longer history of cancer, and a multicountry study found that most patients with advanced cancer preferred to die at home.^8,12^ In Pakistan, a lower-income country, Zafar et al^13^ found that most adult patients with cancer expressed a preference for hospital-based EOL care. A Dutch study found that participants had fixed preferences for either home care or hospital treatment.^14^ A meta-analysis found that most people preferred to die at home,^15^ but a Canadian study at 5 hospitals found that only half preferred dying at home^16^; among South African patients with cancer who were receiving advanced palliative care, approximately 60% preferred to die at home.^17^

There is lack of evidence on EOL care preferences among patients with cancer in China. China is unique owing to low shared decision-making between patients and physicians^18^ and a strong EOL care decision-making relationship between surrogates (mainly family members) and doctors.^19^ As a result, physicians and surrogates operationalize care trade-offs without knowing the preferences of the patients with cancer, especially how much of their lifetime these patients would be willing to lose to attain their palliative care goals.^20^ Surrogates frequently prefer more aggressive cancer treatments and often have greater willingness to pay (WTP) to extend life than do patients.^10,21,22,23^ We examined the stated EOL care preferences of Chinese patients with terminal cancer. This study aimed to provide empirical evidence for clinicians and surrogates on how to inform patients about a full set of preferences for EOL care, improve the quality of patient-centered care, and promote a favorable death for patients.

## Methods

In this survey study, to assess the EOL care preferences of patients with terminal cancer in China, we undertook a discrete choice experiment (DCE) from August to November 2018. DCE is a method used to examine stated preferences over hypothetical alternative scenarios in the health care field.^9,10,14,19,20,24^ Each scenario comprised a number of attributes (days of hospital stay, duration of extended life, quality-of-life improvement, adverse treatment events, place of death, and out-of-pocket expenses), and each attribute had different levels. Willingness to pay and the probability analysis, or uptake rate, were based on the discrete choice model. All patients provided oral informed consent, and the study was approved by Shandong University’s Ethics Committee. The study followed the American Association for Public Opinion Research (AAPOR) reporting guideline. Data analysis was conducted from October 2020 to March 2021.

### Identification of Attributes and Levels

Identification of attributes and their levels is important for ensuring the validity of DCE. We retrieved relevant EOL care attributes and their levels from the extant literature.^5,6,9,10,24,25,26^ The levels established for the attributes of hospitalization days and out-of-pocket costs were based on previous retrospective studies of patients with cancer.^5,6^ The attributes were then ranked, categorized, and reduced through interviews with 10 patients with cancer and 2 experts in the field of palliative care. The specific attribute levels in the DCE design were chosen to ensure trade-offs between tasks and were refined through data from a pilot survey conducted in July 2018 among 20 patients with cancer. Descriptions of the attributes and levels are given in Table 1.

**Table 1.** Discrete Choice Experiment Treatment Attributes and Levels

Attribute and level	Description
Inpatient time spent in hospital	
0	<7 d
1	7-10 d
2	11-30 d
3	>30 d
Extension of life	
0	4 mo
1	6 mo
2	10 mo
3	16 mo
Quality of life associated with treatment^a^	
1	Low (score, 4)
2	Moderate (score, 6)
3	Good (score, 8)
4	Very good (score, 10)
Rate of adverse reactions	
0	None (0%)
1	Low (10%)
2	Moderate (50%)
3	High (90%)
Place of death	
In hospital	Dying in a hospital
At home	Dying at home
Out-of-pocket cost^b^	
1	$1512
2	$6050
3	$12 100
4	$21 174

^a^ Scored on a scale of 1 to 10, with higher scores indicating better quality of life.

^b^ Based on a currency exchange rate of 6.6118 yuan to $1.00 in 2018.

### Survey Design

Based on the pilot survey, our final questionnaires were revised and the phrasing and question layout were improved. To promote survey accuracy, the DCE started with a general description and an illustrative example of a simplified choice set to familiarize the respondents with the choice tasks.^27^

### Study Sample

From 640 hospital medical records from patients with cancer that fit the selection criteria at a 3A level hospital in Shandong Province, we randomly selected 188 patients with terminal advanced cancer at their EOL. Five respondents dropped out, leaving a sample of 183 patients and a 97.3% response rate. Patients were surveyed face-to-face by trained interviewers between August and November 2018. Previous studies found that projection bias may be associated with patients’ decision-making^28^; patients with serious diseases may make decisions based on their current suffering,^28^ and serious health conditions are associated with high preference stability.^29^ Furthermore, older people have been found to more accurately predict their emotional responses and to display more stable preferences than younger people.^30^ Therefore, the sample inclusion criteria were (1) age older than 50 years; (2) diagnosis of stage III or IV cancer; (3) receipt of aggressive treatment, defined by hospitalization during which interventions included surgery, radiotherapy, chemotherapy, and/or targeted therapy^31^; and (4) no cognitive impairments. Based on the Orme equation, the minimum sample size needed was 63.^32^ The sample size was 183 patients, suggesting that the effects of all attribute levels could be accurately estimated. In total, 2928 observations composed the database.

### Statistical Analysis

Using Stata, version 15 (StataCorp LLC), a D-efficient partial profile design assessed estimates of the preference parameters with maximal precision. Sixteen hypothetical tasks, each with 2 alternative choices, were created; however, to reduce the cognitive burden on respondents, these 16 tasks were randomly divided into 2 questionnaire versions. As shown in the eTable in the Supplement, each questionnaire included 8 choice tasks, for which respondents were asked to choose their preferred treatment from 2 alternatives. We conducted validity tests in which respondents answered 1 choice task twice. In addition to DCE preferences, the questionnaires requested information on respondents’ demographic characteristics, including sex, age, location (rural or urban), educational attainment, employment status, income level, cancer type, and cancer stage.

Based on the bayesian and Akaike information criteria,^33^ a mixed logit model was used to estimate patient preferences. All attribute levels were dummy coded except out-of-pocket costs, which was a continuous variable. Mixed logit models that address the problems of preference heterogeneity and independence from an irrelevant hypothesis were used to analyze preferences, with individual utility estimated by length of inpatient hospital stay, duration of extended life, quality of life, adverse events, out-of-pocket medical costs, and place of death (eAppendix in the Supplement).

The WTP for a given change in an EOL care scenario was defined as the amount of money (or money equivalent) that would represent an individual’s marginal payments for the changed attribute levels in a new alternative scenario.^25^ Willingness to pay was estimated from the mixed logit model by comparing the coefficients of the attribute level with that of costs (eAppendix in the Supplement).^34^ The WTP estimates were calculated using the nlcom procedure in Stata, version 15. The probability of choosing a given scenario was assessed using the mixed logit model estimates (eAppendix in the Supplement). *P* values were 2-sided, and the level of significance *P* = .05.

## Results

### Respondent Characteristics

Table 2 presents the demographic characteristics of patients with terminal cancer. Among the 183 respondents with terminal cancer, the mean [SD] age was 61 [8.4] years; most were male (128 [69.8%]), lived in rural areas (130 [71.1%]), had more than 6 years of education (123 [67.2%]), and had a monthly income less than $454 (133 [72.7%]). In terms of cancer types, 98 patients (53.6%) were diagnosed with urologic cancer (kidney, bladder, or prostate), 36 (19.7%) with digestive system cancer (gastric, colorectal, or liver), and 30 (16.4%) with lung cancer. Ninety-three patients (50.8%) had stage III cancer.

**Table 2.** Characteristics of the Study Sample

**Variable**	**Respondents, No.** (**%) (N = 183)**
Sex	
Female	55 (31.2)
Male	128 (69.8)
Age, y	
50-59	72 (39.3)
60-69	82 (44.8)
≥70	29 (15.9)
Marital status	
Married	172 (94.0)
Widowed, unmarried, or divorced	11 (6.0)
Location	
Urban	53 (28.9)
Rural	130 (71.1)
Educational level	
Low (≤6 y)	60 (32.8)
Medium (6 to ≤9 y)	81 (44.3)
High (>9 y)	42 (22.9)
Monthly income tertile^a^	
1 (≤$151)	74 (40.4)
2 ($151-$454)	59 (32.2)
3 (>$454)	50 (27.4)
Basic medical insurance	
Yes	181 (98.9)
No	2 (1.1)
Cancer type	
Lung	30 (16.4)
Digestive (gastric, colorectal, or liver)	36 (19.7)
Urologic (kidney, bladder, or prostate)	98 (53.6)
Other	19 (10.3)
Cancer stage	
III	93 (50.8)
IV	90 (49.2)

^a^ Based on a currency exchange rate of 6.6118 yuan to $1.00 in 2018.

### Estimation of Preferences and Their Heterogeneity

Table 3 presents the results of the mixed logit model. All covariates were significant except hospitalization days (vs less than 7 days) (7-10: β, −0.32 [95% CI, −0.75 to 0.10]; 11-30: β, −0.12 [95% CI, −0.52 to 0.28]; >30: β, 0.34 [95% CI, −0.11 to 0.79]) and a 10% chance of adverse events (vs 0%) (β, −0.33; 95% CI, −0.83 to 0.17). Based on the preference weight in the mixed logit model in Table 3 and the WTP in Table 4, extending life by 10 months (vs 4 months: β, 1.63; 95% CI, 0.81-2.44) and a better quality of life (very good vs poor: β, 1.79; 95% CI, 0.96-2.62) were the most important attributes to patients, followed by out-of-pocket costs, adverse events, and place of death. Patients’ preferences for moderate increase in survival time, better quality of life, death at home, and lower out-of-pocket costs were significantly associated with their choices between treatment models. Patients were more likely to choose to die at home than in a hospital (β, 0.41; 95% CI, 0.03-0.79; SD, 0.34); the SDs in the distribution of the parameters in Table 3 show heterogeneity in patient choice.

**Table 3.** Mixed Logistic Regression Models of Patient Preferences^a^

Variable	β (95% CI)	SE	*P* value	SD	*P* value
Cost	–6.97 × 10^−06^ (–0.0 to –2.80 × 10^−06^)	2.12 × 10^−06^	<.001	NA	NA
Length of hospitalization, d					
<7	1 [Reference]	NA	NA	NA	NA
7-10	−0.32 (−0.75 to 0.10)	0.22	.13	1.15	.02
11-30	−0.12 (−0.52 to 0.28)	0.21	>.99	0.53	.32
>30	0.34 (−0.11 to 0.79)	0.23	.13	1.36	<.001
Life extension, mo					
4	1 [Reference]	NA	NA	NA	NA
6	1.48 (0.77 to 2.19)	0.36	<.001	1.01	.04
10	1.63 (0.81 to 2.44)	0.41	<.001	2.38	<.001
16	1.27 (0.75 to 1.79)	0.27	<.001	0.51	.33
Quality of life					
Poor	1 [Reference]	NA	NA	NA	NA
Moderate	1.29 (0.58 to 1.99)	0.36	<.001	2.25	<.001
Good	1.13 (0.61 to 1.65)	0.27	<.001	0.36	.33
Very good	1.79 (0.96 to 2.62)	0.43	<.001	2.48	<.001
Rate of adverse events, %					
0	1 [Reference]	NA	NA	NA	NA
10	−0.33 (−0.83 to 0.17)	0.25	.19	1.0	.004
50	−0.57 (−1.05 to −0.10)	0.24	.02	0.45	<.001
90	−0.49 (−0.92 to −0.08)	0.22	.02	1.22	.24
Place of death					
Hospital	1 [Reference]	NA	NA	NA	NA
Home	0.41 (0.03 to 0.79)	0.20	.04	0.34	.03

^a^ Model fit data: 2928 observations; 183 respondents; probability, χ^2^ = 0.000; likelihood ratio, χ^2^ (13) = 120.25; Akaike information criterion, 1717.08; bayesian information criterion, 1878.59.

**Table 4.** Willingness to Pay for Level Changes of Specific Attributes

Attribute	Willingness to pay (95% CI), $^a^
Length of hospitalization, d	
<7	1 [Reference]
7-10	–7062 (–23 348 to 2710)
11-30	–2655 (–11 954 to 8986)
>30	7384 (–2928 to 22 034)
Life extension, mo	
4	1 [Reference]
6	32 119 (15 807 to 77 202)
10	35 308 (17 745 to 80 279)
16	27 572 (16 389 to 58 027)
Quality of life	
Poor	1 [Reference]
Moderate	27 963 (14 289 to 58 252)
Good	24 513 (13 314 to 53 798)
Very good	38 854 (19 468 to 95 096)
Rate of adverse events, %	
0	1 [Reference]
10	–7166 (–28 305 to 3591)
50	–12 414 (–32 269 to –2300)
90	–10 791 (–27 110 to –2000)
Place of death	
Hospital	1 [Reference]
Home	8860 (621 to 26 474)

^a^ Based on a currency exchange rate of 6.6118 yuan to $1.00 in 2018.

### Willingness to Pay

Table 4 shows the WTP for level changes in specific attributes. We defined a base EOL treatment choice set: less than 1 week of inpatient days, 4 months of life extension, poor quality of life, no adverse events, and death in a hospital. Patients’ WTP for very good quality of life was $38 854 (95% CI, $19 468-$95 096). The WTP for a 6-month life extension was $32 119 (95% CI, $15 807-$77 202), for a 10-month life extension was $35 308 (95% CI, $17 745-$80 279), and for 16-month extension was $27 572 (95% CI, $16 389-$58 027). The WTP for a lower rate of adverse events (90% rate: –$10 791 [95% CI, –$27 110 to –$2000]; 10% rate: –$7166 [95% CI, –$28 305 to $3591]) showed that patients were willing to pay more for treatment with fewer adverse events. They also were willing to pay $8860 (95% CI, $621 to $26 474) to die at home rather than in a hospital. Based on the WTP results in Table 4, the maximum WTP for the best EOL care scenario (a 10-month life extension, very good quality of life, no adverse events, and death at home) was $83 022.

### Probability of Uptake

The Figure presents EOL care uptake probabilities. We defined the base EOL care scenario as $1512 in costs, less than 1 week of inpatient days, a 4-month life extension, poor quality of life, no adverse events, and death in a hospital. The base EOL care scenario was no change in probability. When the quality of life improved from poor to very good, the uptake rate increased by 61.6%, and when life extension increased from 4 months to 10, the uptake rate increased by 57.2%. The uptake increased by 12.5% when the place of death changed from hospital to home. However, it decreased by 31.4% when the costs increased to $21 174. As detailed in the eFigure in the Supplement, the estimated uptake of the optimal EOL care scenario (10-month life extension, very good quality of life, no adverse events, cost of $1512, and death at home) was 91.0%.

**Figure.**
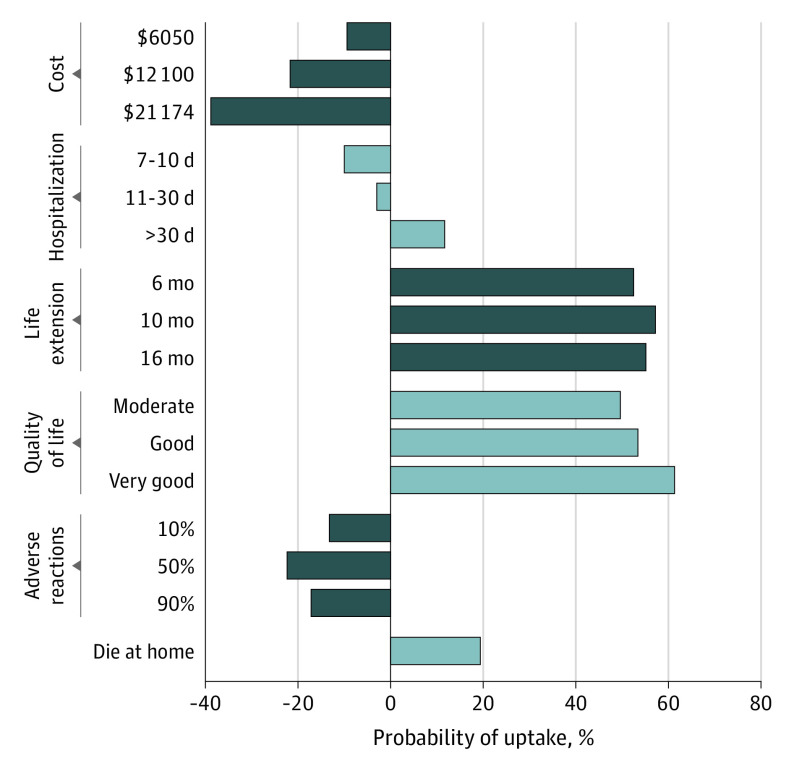
Changes in Probability of Individuals’ Uptake of End-of-Life Care Cost was based on a currency exchange rate of 6.6118 yuan to $1.00 in 2018.

## Discussion

Our study showed that patients with terminal cancer at the EOL preferred a higher quality of life, moderate life extension, less out-of-pocket cost, and death at home. Patients had a greater WTP for improving quality of life from a poor to a very good level than for extending life by half of an additional year or by 1 additional year.

Our study found that the highest quality of life was patients’ highest priority, with patients willing to pay the highest amount ($38 854) to obtain enhanced quality of life rather than a life extension, although there were overlapping 95% CIs (Table 4). When life prolongation increased from 6 months to 10 months, the WTP increased from $32 119 to $35 308, which was higher than the WTP for extending life by 16 months. These results present trade-offs between life extension and quality of life among patients with terminal cancer that are consistent with previous research.^8,11,20^ Waller et al^8^ found that Australian inpatients preferred EOL care that reduced pain and discomfort as much as possible, even if it meant not living longer. Rubin et al^20^ found that 86.7% of US hospitalized patients who were seriously ill would trade at least 1 year of a 5-year life span to avoid a scenario in which they died in the intensive care unit with moderate pain and suffering.

This study’s results contribute to knowledge about cancer treatment preferences for EOL care. As death approaches for Chinese patients with terminal cancer, especially in the last 3 months of life, health resources increase substantially to extend life.^5,35,36^ Our study found that patients had greater preference for comfort-oriented care with a high quality of life than in other studies. In 1 study, only 12.0% of patients with cancer in mainland China received palliative care with a high quality of life at EOL,^5^ but we found that patients preferred care with a high quality of life. This disconnect between patient quality-of-life care and palliative care may reflect a lack of supply of hospice services. First, in China, there are few specialized agencies and a shortage of professional hospice care personnel.^37^ Second, low awareness of hospice care is associated with low acceptance, which is owed to the lack of hospice education and publicity. Third, owing to inadequate shared decision-making about patient care, 35.0% of patients experience medical decisions made by a caregiver.^19^ Family caregivers as surrogates often prefer more aggressive treatment than do patients.^19^

Our results also show that patients with terminal cancer preferred death at home to death in the hospital, and patients were willing to pay more to die at home. These findings are consistent with those of previous studies.^14,15^ Patients wish to die at home for a complex mix of reasons: those who die at home tend to have a higher quality of life and lower medical costs^38^; family members and caregivers of patients who die at home usually have a better quality of life, whereas caregivers of patients who die in a hospital have more severe depression symptoms^39^; and Chinese cultural values may have a role in the attitude toward death and the place of death. Influenced by the traditional Chinese cultural practice of home burial, many people may want to return to their hometown at the end of their life. In 1 study, contrary to patients’ stated preferences, 62.4% of patients with cancer living in urban areas died in a hospital.^40^

We found that patients had preferences for minimizing out-of-pocket expenses, which is consistent with some previous studies.^9,10,12^ A retrospective study of 792 patients with cancer found that patients faced with out-of-pocket costs of more than 50% of their medical expenses could have catastrophic health expenditures forcing many patients into poverty.^5,6^ In the current study, the number of hospitalization days was not a statistically significant attribute. Previous research found that patients with a longer length of hospital stay tended to incur higher medical costs.^5,6,41^ We speculate that the reason that the length of stay did not have a significant association with patient preferences in our study might have been because there was no direct positive correlation between medical costs and hospitalization days in patients’ minds. Of all patients in this study, 98.9% had coverage with one of China’s insurance schemes. The reimbursement rate for hospitalization expenses in China exceeded 55%,^42^ with China’s medical insurance covering the daily hospital bed costs. With patients’ inpatient daily bed costs covered by insurance, patients may not have considered that the actual number of hospital inpatient days affects the final medical costs. The reasons for this finding require further research.

### Limitations

This study has limitations. First, DCEs may not represent all complex real-life EOL care choices given the limited number of attributes and levels. Because we only included patients with cancer who were older than 50 years and were hospitalized, the results may not be representative of patients with cancer in all age groups. Future studies should explore the care preferences of adolescents and young adults. Although this study was adequately powered by the 183 patients, the sample size suggests our results may not be generalizable to China overall. Future studies should expand the sample size appropriately. Also, with larger sample sizes, the influence of other demographic characteristics, such as social status and educational level, on preferences should be further explored. Most patients were diagnosed with urological, digestive system, or lung cancer, with other cancers underrepresented. Additional studies of other types of cancers should be undertaken. Considering most surrogates prefer life-extension treatments,^19^ a preference examination for family caregivers should be undertaken.

## Conclusions

The results of this survey study suggest that a greater focus on improving quality of life and supporting death at home would be consistent with EOL preferences of patients with terminal cancer in China. The findings suggest that physicians and surrogates should ask about patients’ preferences for terminal cancer care, better inform patients of their EOL care choices, and provide guidance for improved EOL outcomes.

## Notes

Supplement. **eAppendix.** Equations in the Methods Section **eTable.** An Example of a Choice Task **eFigure.** Preferences for End-of-Life Care Under Various Potential Scenarios
