# Supplementary material for: Preferences for End-of-Life Care Among Patients With Terminal Cancer in China
Source: JAMA Netw Open. 2022 Apr 25;5(4):e228788. doi: 10.1001/jamanetworkopen.2022.8788 (PMC9039770; doi:10.1001/jamanetworkopen.2022.8788)
Supplement: Supplement. — eAppendix. Equations in the Methods Section eTable. An Example of a Choice Task eFigure. Preferences for End-of-Life Care Under Various Potential Scenarios [file jamanetwopen-e228788-s001.pdf]

Supplementary Online Content

Leng A, Maitland E, Wang S, Nicholas S, Lan K, Wang J. Preferences for end-of-life care among patients with terminal cancer in China. *JAMA Netw Open*. 2022;5(4):e228788. doi:10.1001/jamanetworkopen.2022.8788

eAppendix. Equations in the Methods Section
eTable. An Example of a Choice Task
eFigure. Preferences for End-of-Life Care Under Various Potential Scenarios

© 2022 Leng A et al. *JAMA Network Open*.

eAppendix: Equations in the methods section

$$U_{ijs} = \beta_0 + \beta_1 \mathrm{cost}_{ijs} + \beta_2 inpatient1_{ijs} + \beta_3 inpatient2_{ijs} + \beta_4 inpatient3_{ijs}$$
$$+ \beta_5 extendlife1_{ijs} + \beta_6 extendlife2_{ijs} + \beta_7 extendlife3_{ijs} + \beta_8 qol1_{ijs} + \beta_9 qol2_{ijs}$$
$$+ \beta_{10} qol3_{ijs} + \beta_{11} \mathrm{AR}1_{ijs} + \beta_{12} AR2_{ijs} + \beta_{13} AR3_{ijs} + \beta_{14} \mathrm{hom}e + \varepsilon_{ijs} \qquad \text{(equation 1)}$$

where U_{ijs} is the utility of respondent i for alternative scenario j in the choice tasks (here j = 1, 2; s = 1,..,8), β is a parameter vector relating attribute values and utility levels and ε_{ijs} is error of utility.

$$WTP_i = -\frac{\beta_{\mathrm{i}}}{\beta_{\mathrm{cos}t}} \qquad \text{(equation 2)}$$

$$P_i = \frac{e^{\beta' x_j}}{\sum e^{\beta' x_j}} \qquad \text{(equation 3)}$$

© 2022 Leng A et al. *JAMA Network Open*.

eTable. An example of a choice task

Treatment Characteristics	Treatment A	Treatment B
Number of days you have to stay in hospital	10 days	less than 7 days
Number of days to extend life	10 months	4 months
Quality of life improved by the treatment	Very good	Bad
Side effect of the treatment	Moderate	No
Your out-of-pocket costs in receiving the treatment	140,000RMB[a]	80,000RMB[b]
Place of deathplace	In hospital	At home
**Your choice**		

[a] 80000RMB=US$12,100; [b] 140000RMB=US$21,174

Based on a currency exchange rate of the 6.6118 yuan to US$1.00 in 2018.

© 2022 Leng A et al. *JAMA Network Open*.

eFigure. Preferences for end-of-life care under various potential scenarios

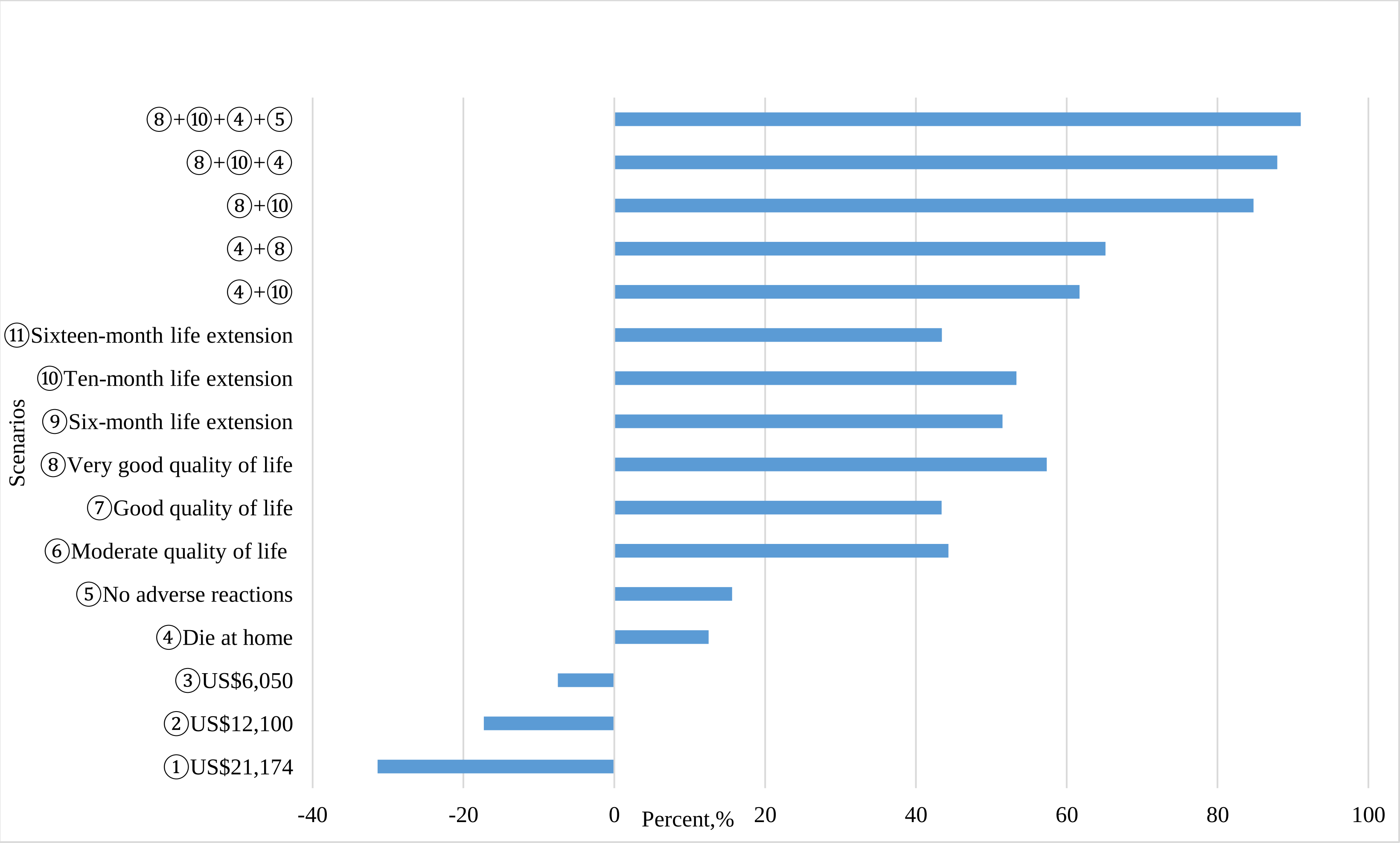

© 2022 Leng A et al. *JAMA Network Open*.
